# The Making of a Modern Hospital

**Published:** 1911-11-18

**Authors:** 


### THE MAKING OF A MODERN HOSPITAL.

### V.—Where Should the Hospital be Placed?

One of the most important points which the architect and contractors of the modern hospital have to weigh is the question of its position. In other words, Where should it be situated?

### Hospital Sites.

It is not necessary for us, at this stage, to go into the interesting question of hospital sites in so far as subsoil, foundations, and geological problems are concerned. This large matter may more suitably be discussed at a later stage. For the present we confine ourselves, then, entirely to the question of environment, and this centres itself mainly round the decision whether the institution should be built in a rural or urban area.

### Urban *v.* Surburban Hospitals.

In considering this question certain points which are self-evident demand attention. Large hospitals are obviously designed for a large hospital population, such as is to be found in big cities. A small community does not stand in need of such an institution, and is better served by a cottage hospital of less than one hundred beds. But it is still the question whether a large hospital, serving a large population, ought to be placed in the immediate centre of that population or whether it ought to be moved to a rural site outside the boundaries of the town or city which it caters for. The older hospitals have all, or nearly all, been placed in central positions. In London, for example, we see the large hospitals grouped round a central area; in Vienna and Paris the same central grouping has been adopted. The Belgian report on hospital construction gives admirable maps showing the disposition of hospitals in the principal cities, from which it is clear that in general the favourite method was to place the institutions more or less centrally. Of late years, however, the tendency has been to build the hospitals on the confines of the large towns, in suburban or semi-rural areas.

### Some Reasons for Changing Hospital Sites.

The main reasons for this change have been the high value, increasing every year, of city sites, the increased facilities for transporting patients to outlying centres with modern quick-moving conveyances, and the increasing desire on the part of the institutions themselves to be as much as possible independent of outside sources of supply. In considering the question one has also to bear in mind the important points in connection with sanitation and to pay some attention to the statistics emanating from the different classes of hospitals. When we take a city such as Madrid, which possesses no suburban hospital, but has two large centrally situated institutions comprising nearly 1,500 beds between them, we find that the authorities are averse from building a new hospital in the suburbs on the ground that they see no necessity for a change since the mortality results are excellent in the two institutions they already possess. In London there is the St. George's Hospital, which occupies a very valuable site in a residential area, and King's College Hospital, which is situated in an area where the ground rent is equally high, and where air space is much curtailed by the surrounding buildings. The authorities of the latter institution decided to remove their hospital to a suburban site, and the new King's College Hospital at Camberwell is the result. With regard to St. George's, it is argued that a change of site will be in the interests of the institution, since the high value of the site which it now occupies will enable the authorities to erect a modern and up-to-date institution in a suburban district where it can be made easily accessible to patients.

### Mortality Figures.

Let us see what the mortality statistics show in regard to the desirability of moving a central city hospital to a suburban site. Taking first the French hospitals, we find that the surgical mortality of the Paris hospitals, all more or less centrally placed, varies from 5 to 9 per cent., the medical mortality from 7 to 23 per cent., the mixed mortality from 6 to 18 per cent., while the average length of stay in hospital of a medical and a surgical patient is 22.8 and 38 days respectively. At Havre, where the hospitals are less centrally situated, the surgical mortality is from 3 to 5 per cent., the medical from 10 to 15 per cent., the average length of stay for the medical patients (statistics with regard to the surgical are not available) 35 days.

In the centrally situated hospitals in Germany the average surgical mortality is 6 per cent., the lowest being 4½; the mixed mortality varies from 4 per cent. (Stuttgart) to 17 (Friedrichshain, Berlin); that of the suburban hospitals, as a type of which we may take the new hospital at Pankow, is in general a point lower. The Swiss and Austrian statistics do not show any difference between suburban or rural hospitals and city institutions centrally placed. Those of Norway, Sweden, and Denmark (collected by Cheval) show a very slight difference in favour of suburban hospitals.*

### Surgical Cases in Urban and Suburban Hospitals.

Taking surgical cases only, most authorities agree that there is no appreciable difference in the post-operative mortality statistics between urban and rural hospitals, classing as rural those large institutions which are not placed centrally. One authority permits himself the following observation: "It is to their disadvantage to place operable cases in the country since it is in their interests that the surgeons should be close at hand, and that they should be

* For a tabulation of the available statistics the reader may consult Depage's critical essay, *La Construction des Hôpitaux.*

under the immediate supervision of the latter, and not under that of recently qualified men. It is therefore indispensable that the surgical clinic of a city hospital should be in the city itself. It is, of course, objected that cases of post-operative infection are more frequent in city than in suburban wards, but such a complication can and must be prevented by scrupulous attention to asepsis. At the St. Jean Hospital in the year 1900 almost all the cases operated on suppurated; a year later the percentage of suppuration cases was twenty-five. In 1906 only 3 per cent. of the cases suffered from this complication, and it is relatively easy to show that the percentage of suppurative cases fluctuates in every hospital, whether urban or suburban, coincident with the change of "firms." The results in cases of operation depend entirely not upon the situation of the hospital but upon its organisation. "Similarly," Martin states, "surgeons demand that we should place our hospitals in the country where the patients have better and purer air. We reply to them that their bad results in the city wards are exclusively due to their own mistakes. Suppuration is avoidable, and ought to be avoided. What is wanted is better constructed wards in which the details of strict asepsis can be rigorously carried out." With this dictum most of us who have any experience of surgical work will cordially agree. In a ward where septic wounds are daily dressed, and where dirty patients are admitted, where the sanitary arrangements are defective, and where visitors and convalescents have the opportunity of introducing sepsis, the wonder is not that suppuration does occur but that it occurs comparatively rarely.

### Medical Cases and Infectious Diseases.

With medical diseases the case is slightly different. They do not stand in need of immediate attention from the visiting physicians, and in certain conditions—notably in the case of infectious diseases—it is better that they should be accommodated in suburban institutions.

With regard to infectious diseases the comparison between an urban central hospital and a suburban hospital is slightly in favour of the former. Take, as an example, Hamburg. Here we have the St. George's Hospital more or less central, and Eppendorf Hospital more or less suburban. At the latter the mortality for typhoid fever cases is 13 per cent.; for the former 16 per cent. (we neglect decimals); for diphtheria it is 11 and 18 respectively; and for scarlet fever 20 and 28 respectively. It is true that in the latter disease St. George's Hospital appears to receive far more serious cases than Eppendorf does—the figures with regard to the percentage of tracheotomies performed prove this—but even then the difference between the two institutions, so far as mortality is concerned, is not very great.

The statistics of our suburban fever hospitals in comparison with those of our infectious hospitals situated in urban areas show the same slight difference. A full discussion of the whole problem will be found in Dr. Vandervelde's interesting memoir on the subject. The main conclusions therein arrived at are that an infectious diseases hospital is preferably placed in a suburban area, that institutions for chronic cases are better placed in rural areas, as also sanatoria, but that so far as a general hospital is concerned the difference in mortality statistics between the urban and suburban institution is so small as to be negligible.

### Further Points for Consideration.

Nevertheless the question must not be approached solely from the side of the statistician. It is possibly true that we have in modern times made too much of the importance of air, light, and other hygienic requirements, and that our whole teaching with regard to the influence which these factors exert on disease will have to be revised. It is as yet too early to speak definitely on these points, especially in view of the fact that the statistics on which this question will have to be judged are not yet available, but certain facts appear to warrant the conclusion that, granted an aseptic environment, a patient suffering from a disease recovers as rapidly in a town as in a village. This may appear to be in direct opposition to the generally accepted teaching, but it is in consonance with the statistical evidence of recovery which we possess.

### The Hospital of the Future.

The main reasons that render it advisable that a large hospital should be placed outside the town are administrative reasons. The ideal, as Sir Henry Burdett has shown, is the hospital city, to which patients can be easily and rapidly transported, and where the best and most up-to-date requirements of medical treatment can be secured with the minimum cost and the maximum of efficiency. In our present social condition that ideal is yet in the dim future, but there are signs pointing to the fact that it is rapidly approaching within measurable distance of realisation. In the meantime we must continue to place our hospitals where they can do the most good.

If questions of finance make it possible to move existing institutions to suburban areas, as in the case of King's College Hospital, such a removal is to be encouraged, care being taken that the place of the old hospital is occupied by a casualty department or a city admission block, since the institution must necessarily draw its inhabitants from the city hospital population. Such removal is all the more necessary in cases where the old hospital stands in a bad environment, where it is encroached upon by out-buildings, and exposed to the noise of outside street traffic.

These are prime considerations which influence the question of situation; they must be weighed and decided on their merits. As a general rule it may be stated that the ideal hospital stands well apart from neighbouring buildings, whether in the city or in the suburban district. For that reason it is a point of importance that it should possess wide grounds so that the patients may enjoy the benefit of pure air and a more or less uncontaminated environment.

